# Western Texas Medical Association

**Published:** 1899-11

**Authors:** 


### Western Texas Medical Association.

SAN ANTONIO, TEXAS, Oct. 30, 1899.

The twenty-third annual meeting of the Western Texas Medical Association was held at San Antonio, Texas, Thursday, October 26, 1899.

Those present were Drs. J. S. Lankford, President; J. V. Spring, B. F. Kingsley, B. E. Hadra, J. H Moore, James Bartlett, A. S. McDaniel, Robt. Dinwiddie, F. M. Hicks, P. Baldessarelli, McP. Barnitz, Rudolph Menger, John S. Turner, Alfred C. McDaniel, A. D. Dupuy, J. P. Oldham, M. L. Graves, J. H. Bindley, T. J. Largen, James H. Bell, R. L. Withers, H. D. Barnitz, Ed. Clavin, Wm. E. Luter, secretary, all of San Antonio, Texas; Dr. E. L. Sharp of Pleasanton, Dr. A. Garwood of New Braunfels, and Dr. T. F. Whiteside of Timpson.

The following program was carried out:

MORNING SESSION, 10 O'CLOCK.

1. A report of a walking case of typhoid fever, resulting in perforation and death. By Dr. Joe H. Reuss, Cuero, Texas.
2. Treatment of Gonorrhœa. By Dr. McPherson Barnitz, San Antonio, Texas.

AFTERNOON SESSION, 2 O'CLOCK.

3. Antepartum Convulsions. By Dr. B. F. Kingsley, San Antonio, Texas.
4. Syphillis of the throat, associated with tuberculosis (with cases). By Dr. Jno. V. Spring, San Antonio, Texas.

5. Tuberculosis of the knee joint, with exhibition of cases. By Dr. Frank Paschal, San Antonio, Texas.
6. Some anatomical and microscopic observations on the *Echinococcus parasite* (cystic tapeworm) and the prevailing epidemic of the Echinococcus disease among the Texas jack rabbits. (Illustrated.) By Dr. R. Menger, San Antonio, Texas.
7. Report of cases.

EVENING SESSION, 8 O'CLOCK.

8. Report of cases, continued.
9. Election of officers.
10. President's address.

A motion was made and duly carried to the effect that the President's address be published*, and that the sense of it be heartily endorsed by the Association. A vote of thanks was tendered Dr. Lankford for the able manner in which he has conducted the affairs of the Association during the past year.

The following officers were elected for the ensuing year: President, Dr. S. Burg of San Antonio; First Vice-President, Dr. J. H. Reuss of Ceuro; Second Vice-President, Dr. A. Garwood of New Braunfels; Secretary and Treasurer, Dr. Wm. E. Luter of San Antonio. Board of Censors, Dr. J. S. Lankford, chairman; Drs. R. E. Moss, C. E. R. King, J. P. Oldham, F. Paschal.

The meeting was a most interesting and instructive one, and the program, as previously announced, was carried out.

After the business session the members retired to the Mahncke hotel, where an elegant banquet was enjoyed.

*The address of Dr. Lankford, the retiring President, and a paper by Dr, J. H. Reuss of Cuero, were received too late for this issue, but will be publishe d in the next.—ED.

